# A spatial typology of energy (in)efficiency in the private rental sector in England and Wales using Energy Performance Certificates

**DOI:** 10.1177/23998083251377128

**Published:** 2025-09-02

**Authors:** Caitlin Robinson, Ed Atkins, Tom Cantellow, Meixu Chen, Lenka Hasova, Alex Singleton

**Affiliations:** 1 School of Geographical Sciences, 1980 University of Bristol, Bristol, United Kingdom; 2 Department of Geography and Planning, 4591 University of Liverpool, Liverpool, United Kingdom

**Keywords:** energy efficiency, private renting, housing inequality, Energy Performance Certificates, k-means clustering

## Abstract

Like many countries globally, the private rental sector in England and Wales contains some of the lowest quality and energy inefficient properties, despite being home to some of the most vulnerable households. We present a new data product that classifies small areas based on the energy (in)efficiency characteristics of private rental properties. Newly available Energy Performance Certificate (EPC) data enables us to analyse detailed energy and housing characteristics for 3.9 million private rentals (∼78.8% of total sector), the most comprehensive dataset of its kind, using k-means clustering. Demographic datasets allow us to explore wider socio-spatial inequalities, and uncertainties associated with granular – but at-times incomplete – EPC data. The classification can be used to evidence how inefficiency is spatially concentrated and fragmented, with a diverse range of energy and housing conditions shaping the everyday lives of tenants.

## Introduction

Energy efficiency is an integral part of a person’s right to a high-quality ambient environment – conditions that allow them to be comfortable, well, and to thrive. It shapes how much energy must be consumed to adequately heat or cool the home (Kerr et al., 2017; Robinson and Williams 2024). Efficiency is a mechanism for tackling some of society’s most pressing challenges, including climate mitigation and adaptation, inequality, and rising energy and living costs (Atkins 2023; Lewis et al., 2020); however, access to good quality housing is highly uneven.

Economic marginalisation, energy insecurity, and housing access are entangled. Without careful thought, policy or technical change can lead to new – or consolidate already-entrenched – patterns of inequity. Housing and energy inequalities are not uniform – variegated between populations and geographies (Carley et al., 2018; Middlemiss 2022) – and the most precarious in society often rely on the lowest-quality housing (Sunikka-Blank and Galvin, 2021).

These dynamics are especially apparent in the private rental sector (Sharma and Samarin, 2022; Robinson et al., 2025). In England and Wales, 5.0 million people rent privately (20.3%), up from 3.9 million in 2011 (ONS 2022a). Since the 2008 Global Financial Crisis, the sector has grown rapidly (Fields and Uffer 2016). Driven by intergenerational and wealth inequalities, and austerity, the private rental sector is increasingly relied upon by some of the most energy vulnerable communities (Ambrose 2015; Petrova and Prodromidou 2019). Private renters are also amongst the lowest earners on average, and spend a higher proportion of income on housing (DLUHC 2023), but quality and efficiency are typically poor (Ambrose 2015).

Detailed understanding of the socio-spatial distribution of efficiency across the private rental sector has been lacking, due in part to a lack of high-resolution energy data (Robinson, 2022; Webborn and Oreszczyn 2019). In this paper we present a data product that addresses this gap, specifically a neighbourhood classification of inefficiency in the private rental sector in England and Wales, derived from analysis of new property-scale Energy Performance Certificates (EPCs). The new detailed evidence from the classification could be used in policy and practice to promote safer healthier living environments whilst meeting climate change targets.

## Data and methods

EPC data appended to Unique Property Reference Numbers – unique identifiers for every address – offers detailed insights into housing characteristics previously impossible to achieve using open-source data (Buyuklieva et al., 2024; Cantellow 2023; Ward et al., 2024). To derive our classification, data is compiled for 3.9 million private rental properties, selecting certificates issued between September 2012 and September 2022 (ODC, 2022). The dataset accounts for ∼78.8% of properties in the sector, due to missingness explained further in Mapping Uncertainty section. Data is most complete for private rentals because a new certificate must be obtained every decade, unlike owner-occupied properties. Important critiques have been levelled at EPC data. They offer estimated rather than actual energy usage, whilst multiple assessors can evaluate the same property and produce different assessments (EAC, 2021; Jenkins et al., 2017; Pasichnyi et al., 2019). However, the certificates remain a widely understood and detailed measure, underpinning government targets (NEA, 2020).

EPC include aggregate scores for efficiency and consumption, as well as physical attributes and efficiency characteristics. Efficiency ratings are estimated between A-G, with A being most efficient (Table 1). Our variables relate to the most intensive energy services – space and water heating.

**Table 1.** Variables selected from EPC data. Data source: ODC (2022).

Variable	Description	Relationship with efficiency
	Variables are provided as a proportion of total private rental properties in Census (Office for National Statistics, 2017) in each OA	
Private rental properties	Private rental properties	Neutral
No mains gas	Without mains gas connection	Negative
Pre-1900	Built prior to 1900	Negative
D or below	EPC rating of D, E, F or G	Negative
F or below	EPC rating of F or G	Negative
Terrace	One of a row of similar houses joined together	Neutral
House or bungalow	-	Neutral
Flat or maisonette	-	Neutral
Inefficient hot water	Efficiency of hot water rated poor or very poor	Negative
Inefficient walls	Efficiency of walls rated poor or very poor	Negative
Inefficient mains heating	Efficiency of mains heating rated poor or very poor	Negative

**Figure 1.**
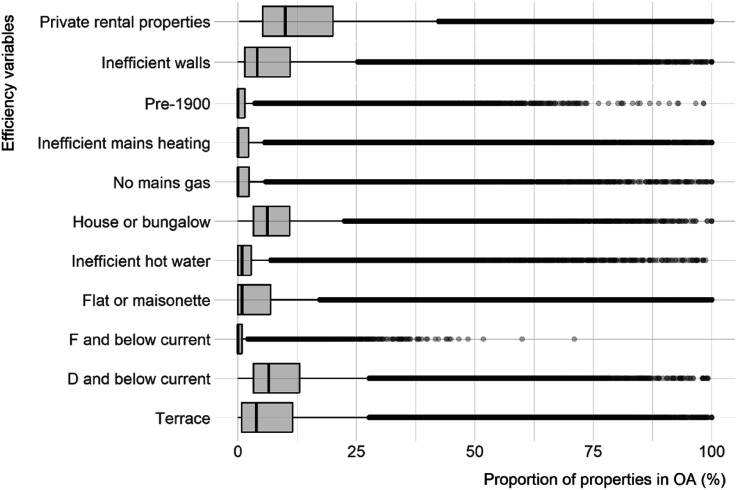
Energy efficiency variables for private rental properties in dataset, based on proportion of total properties in the Census in each OA. Data sources: ONS (2022b) and ODC (2022).

EPC property data is aggregated as counts to Output Areas (OA) (*n* = 188,871) to help overcome gaps in the dataset (ONS, 2022b.). OA contain between 40 and 250 households – as demographically close as possible to street-level – reflecting the diverse built environment in the UK (Arribas-Bel and Fleischmann 2022). OA with less than five properties are removed, retaining 151,890 OA (∼80.4%). Proportions of privately rented properties in OA with various efficiency attributes are computed, comparing the EPC data proportionally to data for privately rented households from the 2021 Census. Variables are standardised using z-scores (Figure 1).

K-means clustering is used to derive the classification. The approach is commonly applied when developing geodemographics, more recently to understand domestic energy inequalities (Chen et al., 2023). K-means iteratively relocates data points between a predefined set of *k* clusters. Each observation belongs to the cluster with the nearest mean. Clusters have a high degree of similarity within, and low degree of similarity between them (Morrisette and Chartier, 2013). We use a MacQueen variation that recalculates cluster centroids each time it iterates over a datapoint, until they converge (MacQueen 1967; Singleton and Longley 2009). Several diagnostics evaluate the final number of clusters: a between-cluster and within-cluster sum of squares statistic. We choose a nine-cluster solution, balancing detail with usability.

## Classification of energy inefficiency in the private rental sector

The new classification of energy inefficiency in the private rental sector contains nine clusters, which are now described in turn based on their density, geography, and intensity of inefficiency. Full cluster descriptions and pen portraits are available at [https://ambient-vulnerability.co.uk/maps/clusters-of-energy-inefficiency-in-the-private-rental-sector-in-england-and-wales/]

**Figure 2.**
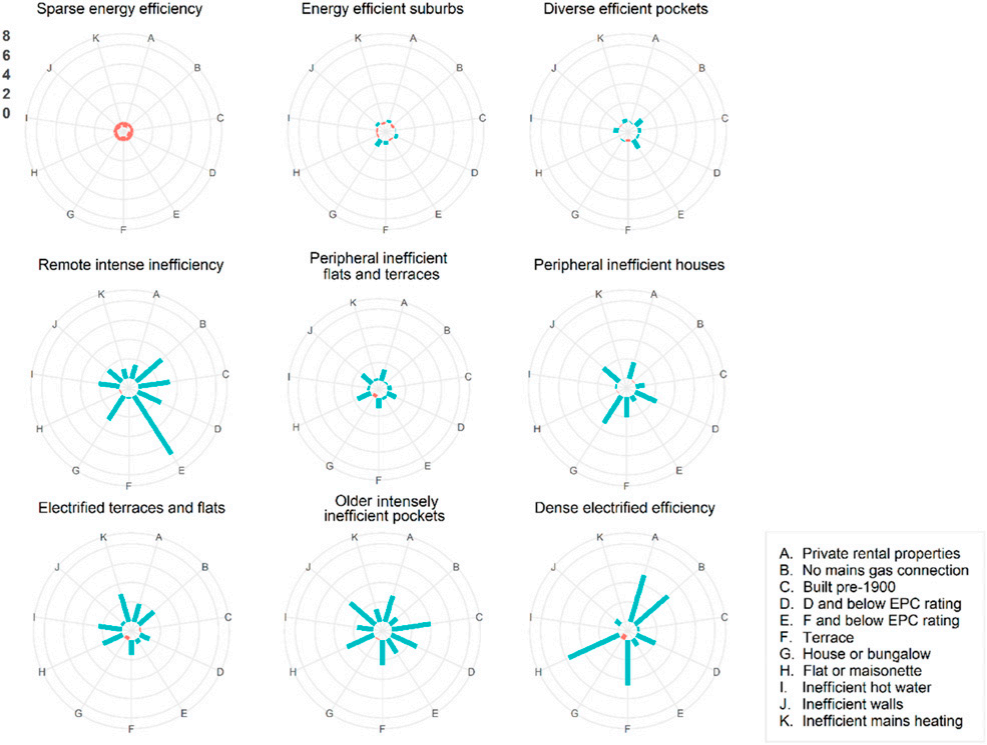
Radial charts to show the centre of the cluster for each underlying variable (as z-scores).

Spatially, private rentals play a dominant role in cities, especially larger urban conurbations, as well as relatively remote rural areas where rentals are often linked to employment. The sector is highly localised, reflected in the range of cluster sizes – the largest contains 89,188 OA, and the smallest 806 OA. Three clusters have high average efficiency – *Sparse energy efficiency* (*n* = 89188), *Energy efficient suburbs* (*n* = 25240), and *Diverse efficient pockets* (n = 13393) – but the proportion of private rentals is low (Figure 2).

**Figure 3.**
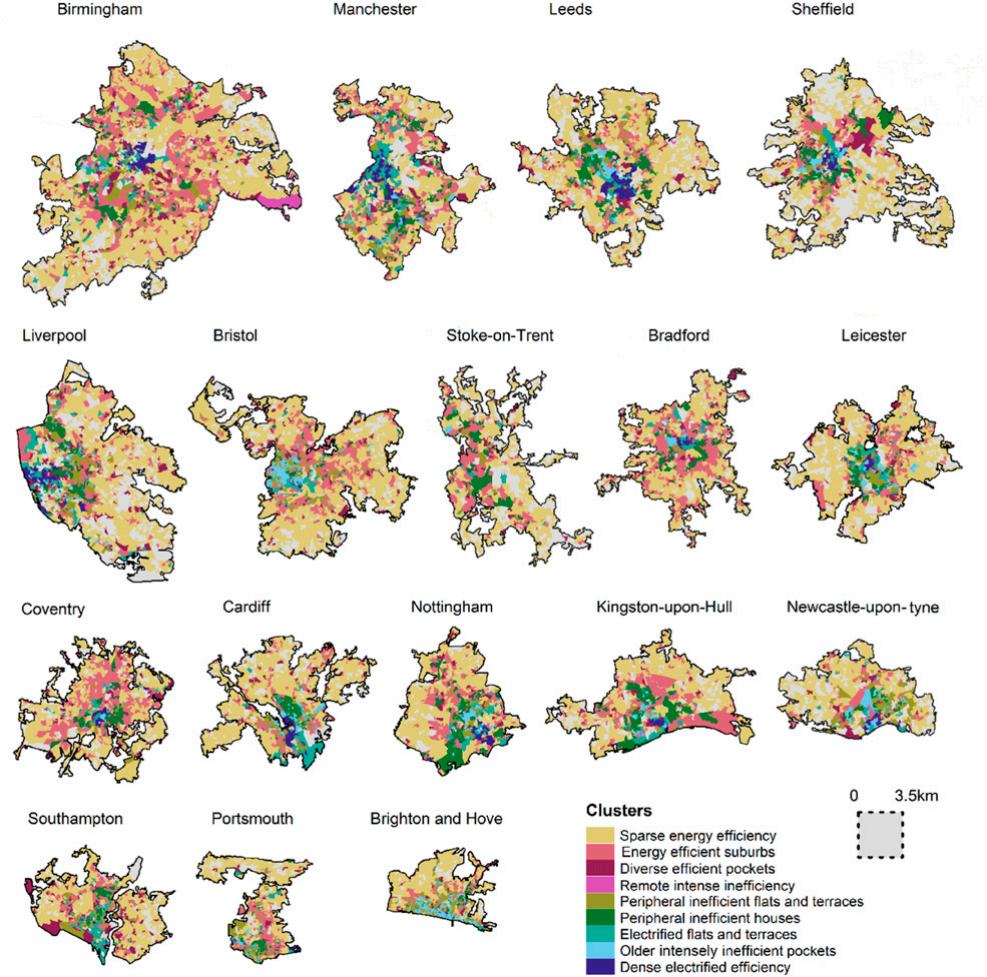
Clusters for major cities. To view the map in detail please visit https://www.ambient-vulnerability.co.uk/. Data source: ONS (2017).

Comparatively, *Dense electrified efficiency* has the highest proportion of private rentals but is the smallest cluster type (n = 806). In these areas, flats typically rely on electricity rather than gas, making energy consumption more expensive (Robinson et al., 2019). These OA concentrate in large cities, especially redeveloped former industrial areas (Lee, 2022) (Figure 3). Although relatively efficient compared to other primarily urban cluster types, many rentals are rated D or below. Similarly reliant on electricity, *Electrified terraces and flats* (*n* = 4223) have a high proportion of private rentals but are spread across urban areas in a fragmented way.

*Peripheral inefficient houses* (*n* = 4888) also have a relatively high number of private rentals, but efficiency is typically low and access to the mains gas is high. *Peripheral inefficient flats and terraces* (*n* = 10721) has a relatively low number of moderately inefficient rentals with access to the gas network. Properties are typically houses or terraces with poor quality walls. Both clusters concentrate on the outskirts of cities and towns, where many properties were built in a similar era.

*Older inefficient pockets* (*n* = 2512) is the least efficient urban cluster type, concentrated in cities and coastal communities (Atkins et al., 2025). OA typically have a high number of private rentals rated D or below, often houses or terraces built pre-1900. Finally, *Remote intense inefficiency* (*n* = 919) has the highest concentrations of properties rated F or below, often off-gas houses built prior to 1900. It has the highest proportion of rentals compared to other relatively rural clusters.

**Figure 4.**
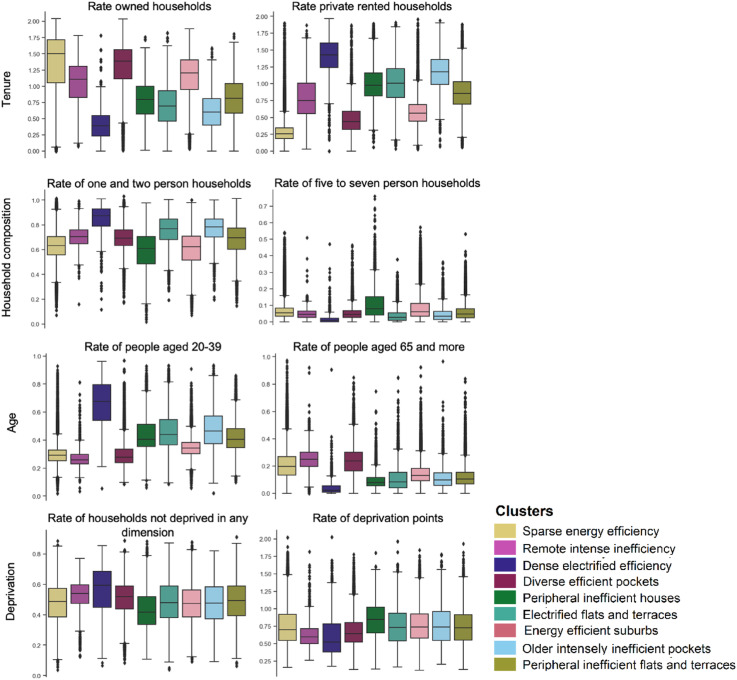
Demographic profile of clusters using rates of persons or households. Data source: ONS (2022b).

The Census 2021 provides insights into the socio-demographic makeup of clusters (Figure 4). *Dense electrified efficiency* consists of a higher proportion of private rental households, with typically younger, full-time professionals or students. *Remote intense inefficiency* and *Diverse efficient pockets* have the most economically inactive population. *Sparse energy efficiency* and *Energy efficient suburbs* have the highest rate of families and children. *Peripheral inefficient houses* have a relatively deprived population and largest household sizes.

## Mapping uncertainty

**Figure 5.**
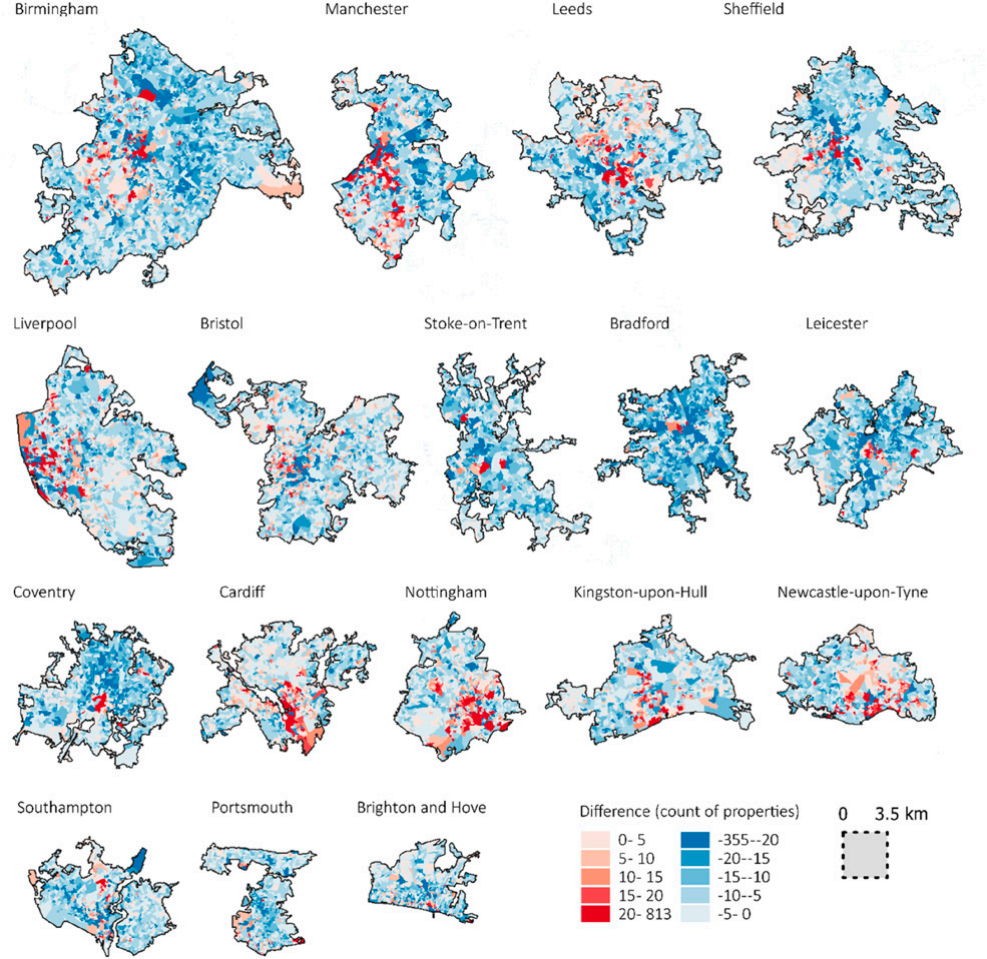
Difference between number of private rental properties in EPC and Census 2021. Red shading illustrates areas with higher number of private rentals in EPC compared to Census. Data source: ONS (2017).

Data gaps, or ‘deserts’, mean certain populations or places are less well represented (D’Ignazio and Klein 2020), but a lack of information can also offer useful insights (Franklin 2022). Approximately 78% of private rentals with an EPC are recorded in the Census (n = 5,023,530), but coverage is geographically uneven (Figure 5). Regionally, Greater London has the highest (83.9%) compared to the West Midlands (71.4%). For individual OA, the greatest difference in count is −355 and +813. A likely explanation for the higher number of private rentals in the Census is a lack of data for Houses of Multiple Occupancy (HMOs),^1^ which are not required to have an EPC but appear in the Census (Atkins et al., 2025). Our classification is not able to account for this sub-sector that often has acute efficiency challenges (Cauvain and Bouzarovski 2016). Additionally, the Census was taken during COVID-19, impacting data quality for renters that temporarily relocated away from city centres (Rowe et al., 2022).

## Applying the classification in policy and practice

The data product can be used to illustrate the diverse range of housing conditions that shape the lives of private rental tenants in different parts of England and Wales. It contradicts the often arbitrary, ‘one-size-fits-all’ use of efficiency in policy and practice. Instead, swathes of cities are characterised by dense, inefficient rentals, whilst some remote rural communities also struggle to access high-quality housing in the sector. Challenges range from young private renters who lack access to the relatively affordable but carbon-intensive gas grid, to older privately rented properties requiring deep and expensive retrofit. This new understanding of inefficiency in the private rental sector could be beneficial to support policy and practice across sectors including housing, health, and climate change.

The classification does not identify a cluster achieving best practice. Yet 5.5% of OA (8,319 of 151,890 OA) are part of the three most inefficient clusters (*Remote intense inefficiency*, *Peripheral inefficient houses*, and *Older intensely inefficient pockets*). In the context of limited public resources these areas might be a focus for targeted policies by local and national government (Moore 2017). However, efficiency measures may also incentivise landlords to increase rents, deepening precarity for tenants. In addition to place-based retrofit (Reames 2016), a universal approach is required to tackle systematic inefficiency in the private rental sector (Bouzarovski and Simcock, 2017).

## Data Availability

To visualise and download the private rental energy inefficiency classification, or to replicate the analysis using code and underlying data, please visit: https://github.com/CaitHRobinson/private-rental-efficiency

## Note

1. In England and Wales, there are an estimated 497,000 HMOs as of 2018 (House of Commons Library 2019). HMOs have five or more occupants who do not form a single household. HMOs are typically less expensive than other private rentals and are more likely to house vulnerable tenants (House of Commons Library, 2019).
